# Aortic Root Dimensions and Pulse Wave Velocity in Young Competitive Athletes

**DOI:** 10.3390/jcm10245922

**Published:** 2021-12-17

**Authors:** Tobias Engl, Jan Müller, Patrick Fisel, Renate Oberhoffer-Fritz

**Affiliations:** 1 Institute of Preventive Pediatrics, Technical University Munich, 80992 Munich, Germany; j.mueller@tum.de (J.M.); patrick.fisel@gmail.com (P.F.); renate.oberhoffer@tum.de (R.O.-F.); 2 Department of Pediatric Cardiology, German Heart Center Munich, 80636 Munich, Germany

**Keywords:** pre-participation screening, aortic root, arterial stiffness

## Abstract

The assessment of aortic root dimensions is a cornerstone in cardiac pre-participation screening as dilation can result in severe cardiac events. Moreover, it can be a hint for an underlying connective tissue disease, which needs individualized sports counseling. This study examines the prevalence of aortic root dilatation in a cohort and its relationship to arterial stiffness as an early marker of cardiovascular risk due to vascular aging. From May 2012 to March 2018, we examined 281 young male athletes (14.7 ± 2.1 years) for their aortic root dimension. Moreover, we noninvasively assessed arterial stiffness parameter during pre-participation screening. Mean aortic diameter was 25.9 ± 3.1 mm and 18 of the 281 (6.4%) athletes had aortic root dilation without other clinical evidence of connective tissue disease. After adjusting for BSA, there was no association of aortic root diameter to pulse wave velocity (*p* = −0.054 *r* = 0.368) nor to central blood pressure (*p* = −0.029 *r* = 0.634). Thus, although a significant proportion of young athletes had aortic root dilatation, which certainly needs regular follow up, no correlation with arterial stiffness was found. It could be suggested that a dilated aortic root in young athletes does not alter pulse waveform and pulse reflection, and thus there is no increased cardiovascular risk in those subjects.

## 1. Introduction

The importance of pre-participation screening in athletes has increased in recent years, especially in children and adolescents, and particularly in the junior squads, where it is now considered a mandatory pre-requisite in many sports. The main purpose of pre-participation screening is to check for pre-existing cardiac and great vessel disease and to assess the risk of developing cardiovascular events [1]. It is well known that vigorous long-term training generates multiple structural and functional adaptions in the heart and the great vessels [2,3], including remodeling of the aortic root [4]. There is no doubt that aortic dissection and rupture are the most serious complications of aortic root enlargement, however, it should be kept in mind that an enlarged aortic root may also cause hemodynamic changes, especially with regard to the pulse wave pattern and central arterial compliance. Aortic root remodeling may change the pattern of the traveling pulse wave negatively to an earlier reflection of the backwards traveling wave, which imposes a greater afterload for the left ventricle resulting in remodeling and dysfunctional buffering [5,6]. There is little knowledge on the prevalence of aortic remodeling in young athletes being still in their growth phase. This study investigates the prevalence of aortic root dilation in young athletes and the association with aortic compliance by means of pulse wave velocity and central blood pressure measurement.

## 2. Materials and Methods

### 2.1. Study Subject

From May 2012 to March 2018, in total 281 young male athletes (14.7 ± 2.1 years) were examined in the context of pre-participation screening in our outpatient clinic for children and adolescents of the Department of Preventive Pediatrics. Within this screening, participants underwent cardiac ultrasound and pulse wave analysis. Their mean training load was 8.2 ± 3.0 h per week for 8.8 ± 2.6 years. Primary sport was soccer (*n* = 250), triathlon (*n* = 10), cross-country skiing (*n* = 8), hockey (*n* = 7), and basketball (*n* = 6).

All participants and guardians gave written informed consent and agreed to the anonymous publication of their data. The study was approved by the local ethical board of the Technische Unversität München (Number: 131/19 S).

### 2.2. Aortic Root Measurement

The examinations were performed by pediatric cardiologists using Prosound α6, α7 (Hitachi Aloka Medical Systems, Tokyo, Japan) and Vivid 7 (GE Healthcare, Horten, Norway) ultrasound machines. The subjects were in a supine position. The aortic root diameter was imaged transthoracically, using a sector transducer in parasternal longitudinal section. In this sectional plane, the left ventricle, aortic root, and proximal portion of the ascending aorta can be assessed. In this position, short cine-loops (video recordings) of 3–5 cardiac cycles were recorded in each case. In parallel, an ECG was recorded.

Measurement of cine loops was performed systematically, according to the American Society of Echocardiography (ASE) guideline [7]. It recommends measuring the diameter in the maximum dimension perpendicular to the course of the aorta at the level of the sinus valsalvae and in end-diastole, i.e., at the beginning of the QRS complex. At this time, the vessel can be visualized in the passive state. In contrast, systolic vessel diameter, as also used in some studies, has the disadvantage of being influenced by stroke volume. The diameter should be measured from the inside of the anterior wall to the outside of the posterior wall of the aortic root (“leading edge-to-leading edge convention”) [7]. In deviation from the current guideline, the diameter was measured in the M-mode instead of the B-mode since many comparative studies on reference values as well as the comparison cohort used in this work also applied this measurement method [8]. To minimize measurement inaccuracies, the mean value from the measurements of three consecutive cardiac cycles was recorded as the measurement result.

### 2.3. Pulse Wave Analysis

Pulse wave velocity as well as peripheral and central blood pressure were measured with an automated oscillometric device (Mobil-O-Graph, I.E.M., Stolberg, Germany) in a supine position after 5 min of rest. The assessment is based on the fact that a part of the aortic pulse wave is reflected at the aortic bifurcation. PWV and central systolic blood pressure (cSBP) were indirectly estimated with the ARCSolver Algorithmus (Austrian Institute of Technology, Vienna, Austria) based on the recorded brachial pulse waves. Hence, the Mobil-O-Graph works fundamentally different from the carotid-femoral PWV measurement, which is currently considered the gold standard among non-invasive measurement methods [9]. Nevertheless, validation and reliability are proved within several studies compared with the invasive intra-aortic catheter measurement method [10]. Another advantage is that the device is fully automated and thus largely examiner-independent [11]. Moreover, unlike carotid-femoral PWV measurement, this measurement method does not rely on the rather inaccurate measurement of aortic length [12].

### 2.4. Data Analysis

All data are presented in mean ± standard deviation (SD) and total numbers and percentages if appropriate. Raw values of aortic root diameters were corrected for BSA and then compared with the reference cohort of Kampmann and colleagues [8]. Values of two standard deviations above the individual reference value were classified as aortic root dilatation. For correlations between aortic root diameter and arterial stiffness parameters (PWV and systolic aortic blood pressure), the partial correlation was used and corrected for BSA and age in all cases. In addition, a general linear model was fitted to the data. That is, a generalization of linear regression by considering more than one independent variable with Bonferroni posthoc test comparing those subjects with aortic root dilation to those without directly adjusting for BSA and age.

Data were analyzed using SPSS 25.0 software (IBM Inc., Armonk, NY, USA). Two-sided *p*-values < 0.05 were considered significant.

## 3. Results

**Figure 1:**
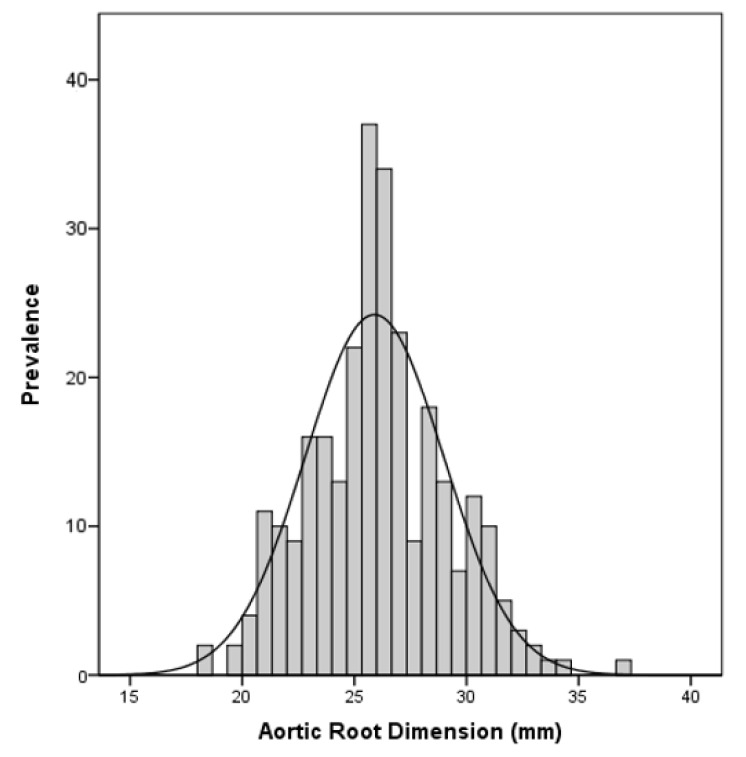
Aortic Root Dimensions in the investigated cohort.

**Figure 2:**
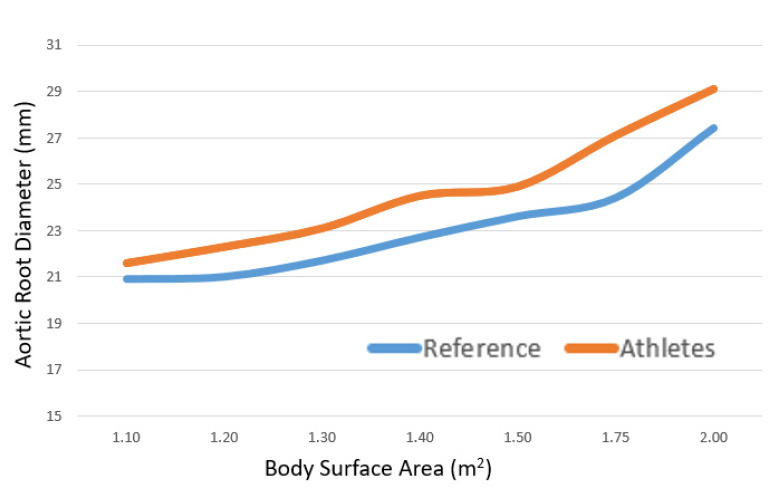
Cross-sectional Aortic Root Dimensions according to body surface area displayed for the athletes and normal healthy reference.

The mean aortic root diameter was 25.9 ± 3.1 mm (Figure 1) and 18 of the 281 (6.4%) of the athletes had aortic root dilation. Athletes had a significantly higher aortic root diameter when compared to a normal reference cohort (athletes: 25.8 mm vs. reference: 23.8 mm; *p* < 0.001) (Figure 2), and there was no association with training hours (*p* = 0.212).

There was no association of aortic root diameter with PWV (*p* = −0.054 *r* = 0.368) nor to central blood pressure (*p* = −0.029 *r* = 0.634) after adjusting for BSA and age. Comparing those 18 subjects with aortic root dilation to those without did not show differences in PWV (dilation: 5.0 ± 0.45 m/s vs. no dilation: 4.9 ± 0.42 m/s, *p* = 0.309) and central systolic blood pressure (dilation: 108.5 ± 12.0 mmHg vs. no dilation: 105.9 ± 10.4 mmHg, *c* = 0.198).

## 4. Discussion

In this study, 6.4% of the screened athletes had aortic root dilation and therewith higher than compared to other reports [13,14,15]. This could explain that the majority of the studies examining prevalence in athlete collectives use the static cut-off values recommended in the 2005 ACC guidelines of 40 mm for men and 34 mm for women [16]. These cut-off values have shown that larger aortic root dimensions are uncommon and probably do not represent the physiological adaptions to exercise but are most likely an expression of a pathological condition and should be monitored [17].

If this study had been based on the same reference values, the prevalence of 2.0% would have been significantly lower. However, a comparison with the fixed reference value of 40 mm makes little sense for various reasons. Children and adolescents have significantly smaller heart diameters than adults [18]. Therefore, maturation is why we have chosen this dynamic adjustment for our young collective (mean age 14.7 years). It should be noted that values of two standard deviations above the norm are not pathological per se. However, they should at least be noted as suspicious for the age of the patient and monitored for syndromes associated with aortic root diseases like Marfan Syndrome and Loeys-Dietz Syndrome [19], which could not be identified in this cohort. That aortic root dilatation likely represents a pathological process and not a physiological adaptation to exercise, which was also concluded in a comprehensive meta-analysis from Iskandar and colleagues [20] because athletes showed only small larger aortic root diameter.

There is good evidence that clinically relevant aortic dilatation is uncommon among athletes in general [13,15,21]. Nevertheless, relevant aortic dilatation is reported in aging endurance athletes, raising the possibility of vascular remodeling in response to long-term exercise [22]. In this report, there was no association with training hours and training load. Three reasons seem reasonable for the absence of this correlation. Since this report measured young athletes, the exposure to training and a possible change in the aortic root may simply be too short to be measurable. Secondly, the survey of the extent of training was based on recall, which is known to be imprecise. Third, the fact that our group consisted of endurance athletes and did not include classic strength athletes could also have played a role here. A research group around D’Andrea [23] was able to show clear differences in young adult athletes with regard to the athletic profile. In their cohort, diameters and stiffness were significantly greater in strength-trained athletes, while aortic distensibility was higher in endurance athletes. Finally, many studies also reported an association with body length, which is not evident from our data.

Considering the above-mentioned aortic remodeling from another perspective—from the cardiovascular, it is known that in young individuals, the aorta is elastic and expands to accommodate the stroke volume during systole and then recoils to stored energy [24]. Stiffening of the aorta and the aortic tree is a key, independent determinant of cardiovascular risk, morbidity, and mortality [12,25] due to earlier reflection of the forwards traveling pulse wave. The pulse wave takes its origin during the ejection of blood into the ascending aorta, thus at the level of the aortic root. Although no correlations were found between aortic root diameters and the stiffness parameters, PWV, and central systolic blood pressure, in this study, it can be speculated that the pulse wave is already altered in its development because the pulse wave arises precisely at the aortic root. At least there is evidence that pulse wave is altered when aortic size is larger at the level of the sinuses of Valsalva [26]. Vriz and co-workers [27] also found that the increment in aortic diameter with age was less when adjusted for aortic stiffness and therefore also recommend taking aortic stiffness measures into account when the increase of aortic diameter is considered.

Decisive research is certainly needed here. Regardless, especially in athletes with abnormal aortic root diameters, the focus should not only be on the risk of aortic dissection but also on whether changes in the pulse wave are present, especially in view of the fact that this methodology is non-invasive, less time-consuming, and inexpensive [28].

## 5. Conclusions

Although a significant proportion of young athletes had aortic root dilatation, no correlation with arterial stiffness was found. It could be suggested that pulse waveform and pulse reflection are not altered by a dilated aortic root, and thus, there is no increased cardiovascular risk in those subjects. However, possible exercise-related triggering of further aortic growth should be followed in serial pre-participation examinations.

## Data Availability

The data is available upon request from the corresponding author.
